# Modularization of biochemical networks based on classification of Petri net t-invariants

**DOI:** 10.1186/1471-2105-9-90

**Published:** 2008-02-08

**Authors:** Eva Grafahrend-Belau, Falk Schreiber, Monika Heiner, Andrea Sackmann, Björn H Junker, Stefanie Grunwald, Astrid Speer, Katja Winder, Ina Koch

**Affiliations:** 1 Technical University of Applied Sciences Berlin, FB VI/FB V, Bioinformatics/Biotechnology, Seestr. 64, 13347 Berlin, Germany; 2 Leibniz Institute of Plant Genetics and Crop Plant Research (IPK), Dept. of Molecular Genetics, Corrensstrasse 3, 06466 Gatersleben, Germany; 3 Brandendenburg University of Technology at Cottbus, Dept. of Computer Science, Postbox 10 13 44, 03013 Cottbus, Germany; 4 Poznań University of Technology, Institute of Computing Science, ul. Piotrowo 2, 60-965 Poznań, Poland; 5 Max Planck Institute for Molecular Genetics, Dept. Computational Molecular Biology, Ihnestr. 73, 14195 Berlin, Germany

## Abstract

**Background:**

Structural analysis of biochemical networks is a growing field in bioinformatics and systems biology. The availability of an increasing amount of biological data from molecular biological networks promises a deeper understanding but confronts researchers with the problem of combinatorial explosion. The amount of qualitative network data is growing much faster than the amount of quantitative data, such as enzyme kinetics. In many cases it is even impossible to measure quantitative data because of limitations of experimental methods, or for ethical reasons. Thus, a huge amount of qualitative data, such as interaction data, is available, but it was not sufficiently used for modeling purposes, until now. New approaches have been developed, but the complexity of data often limits the application of many of the methods. Biochemical Petri nets make it possible to explore static and dynamic qualitative system properties. One Petri net approach is model validation based on the computation of the system's invariant properties, focusing on t-invariants. T-invariants correspond to subnetworks, which describe the basic system behavior.

With increasing system complexity, the basic behavior can only be expressed by a huge number of t-invariants. According to our validation criteria for biochemical Petri nets, the necessary verification of the biological meaning, by interpreting each subnetwork (t-invariant) manually, is not possible anymore. Thus, an automated, biologically meaningful classification would be helpful in analyzing t-invariants, and supporting the understanding of the basic behavior of the considered biological system.

**Methods:**

Here, we introduce a new approach to automatically classify t-invariants to cope with network complexity. We apply clustering techniques such as UPGMA, Complete Linkage, Single Linkage, and Neighbor Joining in combination with different distance measures to get biologically meaningful clusters (t-clusters), which can be interpreted as modules. To find the optimal number of t-clusters to consider for interpretation, the cluster validity measure, Silhouette Width, is applied.

**Results:**

We considered two different case studies as examples: a small signal transduction pathway (pheromone response pathway in *Saccharomyces cerevisiae*) and a medium-sized gene regulatory network (gene regulation of Duchenne muscular dystrophy). We automatically classified the t-invariants into functionally distinct t-clusters, which could be interpreted biologically as functional modules in the network. We found differences in the suitability of the various distance measures as well as the clustering methods. In terms of a biologically meaningful classification of t-invariants, the best results are obtained using the Tanimoto distance measure. Considering clustering methods, the obtained results suggest that UPGMA and Complete Linkage are suitable for clustering t-invariants with respect to the biological interpretability.

**Conclusion:**

We propose a new approach for the biological classification of Petri net t-invariants based on cluster analysis. Due to the biologically meaningful data reduction and structuring of network processes, large sets of t-invariants can be evaluated, allowing for model validation of qualitative biochemical Petri nets. This approach can also be applied to elementary mode analysis.

## Background

Structural analysis of biochemical networks (i.e. metabolic, signal transduction and gene regulatory networks) is a growing field in bioinformatics, especially considering the availability of nearly complete metabolic networks of several organisms [1,2]. Elementary mode analysis [3], extreme pathway analysis [4], and Petri net invariant analysis [5] are established methods to qualitatively analyze biochemical network models. By giving insight into the basic system behavior, these qualitative approaches can be used to check a model for consistency and biologically meaningful behavior, allowing for model validation. The first two methods (elementary mode and extreme pathway analysis) are predominantly applied to metabolic networks [6,7], Petri net theory is additionally applied to signal transduction [8-10] and gene regulatory networks [9,11,12], and combinations of them [13-15]. A detailed review of the use of Petri nets in systems biology is given in the references [16,17], or [18].

Petri net theory is a mathematical formalism, enabling formal and clear representation of biochemical networks at different abstraction levels, as well as their structural analysis. In contrast to the concepts of elementary modes and extreme pathways, Petri nets additionally provide analysis techniques for the computation of static and dynamic network properties [19]. Other strong advantages are the visual representation and animation facilities, which support the intuitive comprehension of the network and provide a useful communication platform between theoreticians and experimentalists. Because of these reasons, we use Petri nets for modeling biochemical networks. All examples in this paper were developed as Petri nets and validated using Petri nets techniques.

The concept of minimal non-negative t-invariants forms the basis for validating biochemical Petri nets. A crucial point in model validation [19,20], is that the net should be covered by t-invariants, and that there should be no t-invariant without a sensible biological meaning. When addressing the second point, the exponentially growing number of minimal invariants in large biochemical networks creates the need for additional concepts and tools, as large numbers of invariants can no longer be handled and interpreted manually.

Modularization techniques [21,22], which automatically decompose complex networks into functional modules, can be applied to facilitate the analysis and interpretation of biochemical systems and their basic behavior. The computation of *maximal common transitions sets *(MCT-sets) [10], which can be read as functional units (i.e. units with a distinct biological meaning), allows for the examination of t-invariants by decomposing a given network into biologically meaningful modules.

A common approach to handling large data sets is cluster analysis, a data mining technique used to group objects of similar kind into respective subsets. Due to the identification of relatively homogeneous subsets, the application of clustering techniques results in a structured and reduced data representation, facilitating the analysis and interpretation of large data sets. To provide a biological analysis of large numbers of elementary modes, Pérès *et al*. [23] elaborated a classification method of elementary modes called aggregation around common motif (ACoM). By grouping elementary modes into classes with similar substructures, this method allows the interpretation of classes of elementary modes to find their biological meaning. To the best of our knowledge, no clustering approaches in the analysis of Petri nets existed until now. In this paper, we demonstrate how this novel application contributes to the analysis of Petri net t-invariants. We introduce and discuss the classification of t-invariants based on cluster analysis by applying different clustering techniques and distance measures to various sets of t-invariants of biochemical Petri net models. After a short introduction to Petri nets and the clustering techniques used, we illustrate and discuss the clustering results in two case studies: a small signal transduction pathway and a medium-sized gene regulatory pathway.

## Methods

### Petri nets

**Figure 1:**
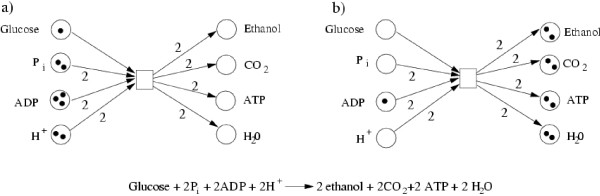
A biochemical example for the Petri net firing rule. The stoichiometric equation represents the conversion of glucose into ethanol, an alcoholic fermentation. The stoichiometric factors correspond to the arc weights in the Petri net. Figure 1a indicates the marking before the firing, whereas Figure 1b corresponds to the marking after the firing.

A Petri net, *N *= (*P*, *T*, *F*, *M*_0_), is a directed bipartite graph with two types of nodes, which are described by the finite sets, *P *and *T *. *Places*, *p *∈ *P*, drawn as circles, typically model the passive part of the network, which in biochemical Petri nets are the chemical compounds (e.g. metabolites or complexes). *Transitions*, *t *∈ *T*, drawn as rectangles, generally stand for the active part (e.g. stoichiometric chemical reactions, complex formation, de-/phosphorylation, de-/activation). The set, *F*, describes the directed arcs between places and transitions and vice versa. In metabolic networks, the arcs are weighted by the stoichiometric factors of the underlying stoichiometric reaction equations, whereas in signal transduction and gene regulatory networks the arc weight is usually set to one at the beginning of the modeling process [10,24], because no stoichiometric equations exist. There are movable objects, *tokens*, which are located on places. They usually correspond to an amount (e.g. a mole) of the chemical compound (e.g. a metabolite). The distribution of tokens over the places is called a *marking*, *M*, representing a certain system state. *M*_0 _describes the initial marking, before any reaction took place. The movement of tokens is defined by the *firing rule*. A transition can fire, or take place, if it is enabled; that is, if the pre-places, also called pre-conditions, carry at least as many tokens as indicated by the weights of the transition's incoming arcs. This means that as many tokens will be removed from the pre-places of the transition as are indicated by the arc weights of its incoming arcs, and as many tokens are put on the post-places as are given by the arc weights of its outgoing arcs (Figure 1). This firing rule is timeless and describes a discrete process. Note that tokens can be produced within the system and removed from the system.

Now, static and dynamic network properties can be computed to analyze the system behavior. In the following, we will focus on these properties, which are essential to our analysis. For a more formal and detailed introduction to Petri nets, see references [25-28], or [29]. We continue with the introduction of system invariants, especially the *t*-invariants [5], which form the basis for all further analyses in this paper.

### T-invariants

**Figure 2:**
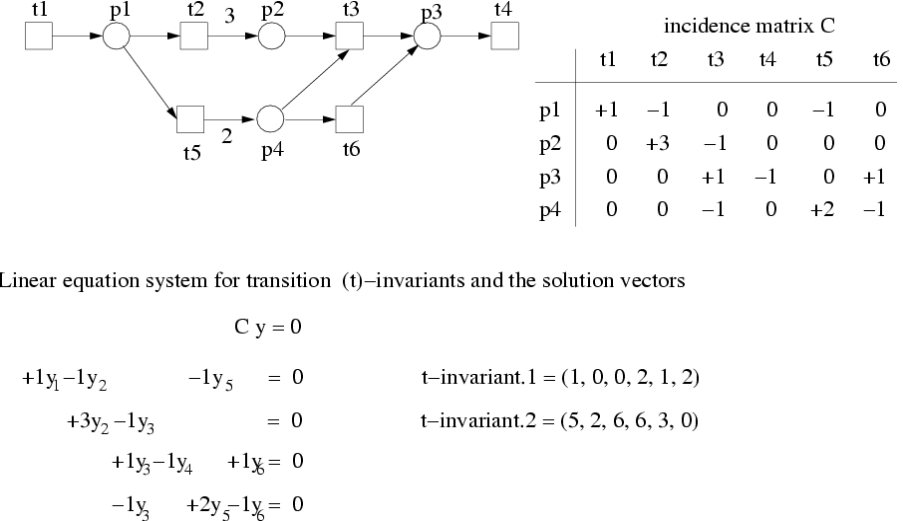
An example of a Petri net with its incidence matrix, the corresponding linear equation system for the computation of t-invariants, and the resulting solution vectors.

A Petri net's incidence matrix corresponds to the stoichiometric matrix in a metabolic network. The incidence matrix comprises the change in token amount for each place when a single transition of the whole network fires (see Figure 2). Based on an incidence matrix, *C*, the linear equation system, *C *· *y *= 0, can be formulated to calculate the t-invariants, *y*. The t-invariants describe the system behavior of the network, e.g. for metabolic networks in the steady state. Only nontrivial, non-negative integer solutions are of interest. Because the non-empty solution space of such linear equation systems is infinite, we want to get a minimal and favorably finite characterization of all solutions. Thus, we are searching for *minimal *nontrivial, non-negative integer solutions.

The elements corresponding to an invariant's non-zero entries are called the *support *of this invariant. To get minimal invariants, the support of one invariant must not be contained in another one, and the greatest common divisor of all entries of the invariant vector is equal to 1. In the following, we write *t-invariants *instead of *minimal t-invariants*. The support of a vector (i.e. of a t-invariant) contains the set of transitions with an entry greater than 0, without any additional information, as, for example, the weight for each transition in the t-invariant. Vectors, containing frequencies of the elements, are called Parikh vectors (for the formal definition see [53]), which are interpreted in Petri net theory as a frequency vector of firing transitions. The solution vectors of the linear equation system give multisets of transitions, which can be interpreted in the context of Petri nets as well as in a biological context.

The Petri net interpretation of t-invariants means that the firing of transitions, indicated by the entries in the Parikh vector formed by t-invariants, reproduces an arbitrary system state, indicated by a token distribution. In biology, the interpretation was introduced by Schuster *et al*. [3], and represents the well-known concept of elementary modes. The correspondence of elementary modes and minimal t-invariants is given by their definitions. The concept of elementary modes is described on the basis of a finitely generated convex cone. Elementary modes represent solution vectors on the surface and in the interior of this cone. The cone is polyhedral, and is given by the corresponding linear inequation system [30]. This concept has mainly been applied to metabolic systems. Here, minimal t-invariants and elementary modes, respectively, are interpreted as the minimal set of enzymes, which represent the chemical reactions or transitions, respectively, which can operate at steady state. T-invariants have also been used for analyzing signaling and gene regulatory networks [8,10,12]. Meanwhile, also elementary modes are applied to the analysis of signaling pathways [31]. Please note that in signal transduction and gene-regulatory networks generally no stoichiometric relations are known, such that the invariant condition cannot be interpreted as the steady state known in biology. Since the set of minimal t-invariants is a generating system for the solution space, all solutions (not only the minimal t-invariants) can be computed by a linear combination of the minimal ones.

According to our validation criteria for biochemical Petri nets [19,20], this also means that each transition must be contained in one of the minimal t-invariants. This property is called *covered by t-invariants*, or for short, *CTI*. T-invariants define subnets, and minimal t-invariants define self-contained subnets, which are always connected. These subnetworks describe biological pathways in the network, and each should be checked for its biological meaning to validate the model. The occurrence of a biologically senseless or unknown pathway can indicate a modeling error or a new pathway detection, which can induce further experiments. Unfortunately, the number of t-invariants can grow exponentially. For larger and more complex biochemical networks, the number of t-invariants can be in the thousands or more. In this case, our clustering approach can help to interpret these large sets of t-invariants.

### Pathways and subpathways

A *pathway *is a part of a biochemical network (i.e. a subnetwork) with a special biological meaning. In graph theory, it corresponds to a subgraph of the whole graph. A pathway is not necessarily linear; it may contain branches and joints. Well-known biochemical pathways are, for example, the glycolytic pathway or the pentose phosphate pathway. Consequently, a *subpathway *is again a part of a pathway, but not necessarily related to a whole biological functional unit.

### Cluster analysis

Cluster analysis comprises a range of methods for classifying multivariate data into subsets (clusters) based on similarity. By partitioning heterogeneous data into relatively homogeneous clusters, clustering can help to identify the intrinsic grouping in the data set and to reveal the characteristics of some structure in the data. In biology, clustering techniques are applied to many different fields of study, such as ecology, zoology, and molecular biology (e.g. gene expression analysis) [32,33]. A cluster analysis can be seen as a three step process, encompassing the following main steps: (1) selection of a distance measure to compute the distance between all pairs of objects, (2) selection of a clustering algorithm to group the objects based on the computed distances, and (3) selection of a cluster validity measure to identify the optimal number of clusters for interpretation. A detailed description of each of these steps is given below.

#### Distance measures

To assess the similarity between two objects, *o*_*i *_and *o*_*j*_, it is necessary, first, to describe the objects according to some scheme and then choose an appropriate measure to compare the description of the objects. A common method is to describe the objects by the presence or absence of features and to compute the similarity by comparing the binary feature vectors, *χ*_*i*_, *χ*_*j*_, of the objects, {*o*_*i*_, *o*_*j*_}. In our case, the objects considered are the t-invariants, and the feature vectors correspond to the support vectors of these invariants. The *Tanimoto *coefficient [34] has been chosen as the similarity measure. Based on this coefficient, the similarity between two t-invariants, *t*_*i *_and *t*_*j*_, is computed as:

$$s(t_{i},t_{j})=s_{ij}=\frac{a}{a+b+c}$$

where *a *is the number of features present in both objects, *b *is the number of features only present in object, *i*, and *c *is the number of features only present in object, *j*. The pair-wise similarity, *s*_*ij*_, is transformed into a distance, *d*_*ij *_[35], by

*d*_*ij *_= 1 - *s*_*ij*_.

For the clarity of illustration, an example is given below:

$$s(t_{i},t_{j})=s\left(\left(\begin{array}{c} 1\\ 1\\ 0\\ 0 \end{array}\right),\left(\begin{array}{c} 1\\ 0\\ 1\\ 0 \end{array}\right)\right)=\frac{1}{1+1+1}=\frac{1}{3};d_{ij}=1-\frac{1}{3}=\frac{2}{3}$$

Beside the Tanimoto coefficient, several other distance measures (*Simple Matching *[34], *Sum of Difference *[34]) have been tested in preliminary investigations. Supported by an external cluster validation (i.e. the evaluation of clustering results based on the knowledge of the correct classification of objects), the distance measures were validated and compared on the basis of various sets of t-invariants from different types of biochemical Petri nets describing metabolic, gene regulatory, and signal transduction nets. In this paper, only the distance measure showing the best results, the Tanimoto coefficient, is applied to the data. A brief discussion of the other distance measures is given as additional material [see Additional file 1].

#### Clustering algorithms

Having obtained the distance between each pair of objects in the data set, a hierarchical clustering algorithm is applied, which successively merges the objects into binary clusters resulting in an ordered sequence of partitions (i.e. a hierarchical clustering tree or dendrogram). In the dendrogram produced by the clustering algorithm the objects are located at the leaves of the dendrogram, and similar objects occur in proximate branches. The dendrogram can be cut at any level to yield different clustering, i.e. partitions, of the data.

In this paper, three agglomerative hierarchical clustering algorithms (*UPGMA*, *Single Linkage*, and *Complete Linkage*) are applied. In general, an agglomerative clustering algorithm starts with the finest partitioning (singleton clusters), merging the two most similar clusters in each iteration, until all clusters are joined in one cluster. Agglomerative algorithms differ in the way the distance between a pair of two clusters is determined. In UPGMA, the distance between two clusters, *C*_*i *_and *C*_*j*_, is computed as the average of distances, *d*_*ij*_, of all possible pairs of objects with *i *∈ *C*_*i *_and *j *∈ *C*_*j *_. In Single Linkage and Complete Linkage, the distance between two clusters, *C*_*i *_and *C*_*j*_, is the minimum of distances, *d*_*ij*_, or maximum of distances, *d*_*ij*_, respectively, of all possible pairs of objects with *i *∈ *C*_*i *_and *j *∈ *C*_*j *_.

As a fourth method, the *Neighbor Joining *algorithm, a clustering technique widely used for phylogenetic tree construction, is implemented. The Neighbor Joining algorithm, originally introduced by Saitou and Nei [36], and modified by Studier and Keppler [37], successively joins pairs of neighboring objects. To determine two neighboring objects, the average distance of each object to each other object is computed and subtracted from the pairwise distance, *d*_*ij*_, between the objects.

In this paper, the algorithm given by Saitou and Nei [36], which generates an unrooted tree (in phylogeny interpreted as a tree with an unknown evolutionary ancestor), has been modified to build a rooted tree, by assigning a root node in between the two clusters remaining at the end of the clustering process. For a detailed description of clustering techniques, see e.g. [38]. Once the hierarchical clustering tree has been built, we have to decide the most suitable number of clusters.

#### Number of clusters

The prediction of the optimal number of clusters to consider for interpretation, or the decision of where to cut the hierarchical tree, is a fundamental problem in unsupervised classification. To overcome this problem, various cluster validity measures have been proposed to assess the quality of a clustering partition (for review, see [39] and [33]), thus helping to identify the number of clusters that "best" represents the intrinsic grouping of the data. After testing different validity measures (*Silhouette Width *[40], *Dunn*- [41], *Davies-Bouldin*- [42], and *C-index *[43]) in preliminary investigations, the measure showing the best results, the Silhouette Width, is applied to the data. A brief discussion of the other validity measures is given as additional material [see Additional file 2]. The Silhouette Width for a clustering partition is computed as the average Silhouette value over all data samples. The Silhouette value, *S*, for an individual data sample, *i*, is defined as

$$S(i)=\frac{b_{i}-a_{i}}{max(b_{i},a_{i})},$$

where *a*_*i *_denotes the average distance between *i *and all the data samples in the same cluster, and *b*_*i *_denotes the average distance between *i *and all data samples in the closest other cluster (i.e. the cluster yielding the minimal *b*_*i*_). The Silhouette Width is limited to the interval [-1,1] and should be maximized. The shape of the Neighbor Joining dendrogram does not permit the application of the proposed cluster validity measures, as the cutting of the dendrogram at a given hierarchical level does not always result in a partition comprising the sum of all clustered objects. Therefore, the optimal number of clusters for the Neighbor Joining dendrograms is determined based on visual inspection.

#### T-cluster

The proposed clustering approach results in clusters of t-invariants, t-clusters for short. The set of transitions characterizing a given t-cluster (i.e. those transitions that participate exactly in all t-invariants of the t-cluster) define subnets, which can overlap or even contain each other. Due to their distinct biological meaning, these subnets can be read as functional units. These modules, which are defined by the resulting t-clusters, can be used for decomposing a network into biologically relevant functional units. Transitions not contained in any of these modules correspond to functional units, which characterize a subset of t-invariants of a given t-cluster. In general, these functional units correspond to trivial modules, i.e. modules consisting of only one transition.

### Modeling and classification approach

The biochemical pathways used as case studies in this paper are modeled as Petri nets and validated according to Koch and Heiner [19]. In order not to describe the whole modeling process we chose already published models [10,24] and discuss the clustering results here. We consider two different types of biochemical pathways, a signal transduction and a gene regulatory pathway. The computed t-invariants are clustered based on the Tanimoto distance measure and the clustering methods UPGMA, Single Linkage, Complete Linkage, and Neighbor Joining. To determine the optimal number of t-clusters for interpretation, the quality of all clustering partitions of a given dendrogram is determined based on the Silhouette Width, and the partitioning maximizing the validity measure is chosen for interpretation. All models are given as additional material [see Additional file 3].

## Results and Discussion

### Results

In the following, two well-investigated examples, a signal transduction pathway and a gene regulatory pathway, are given to demonstrate the usability of our approach. After a short introduction to the biological background of each pathway, the clustering results are presented and an evaluation of the applied clustering techniques is given.

### Pheromone response pathway in yeast

#### Biological background

The pheromone response pathway of *Saccharomyces cerevisiae *(yeast) is one of the best understood signal transduction pathways in eukaryotes [44]. Two haploid yeast cells of opposite mating types are able to mate and fuse into one diploid cell. For this purpose, the response of one cell to the presence of a cell of the opposite mating type is triggered by a secreted peptide mating pheromone binding to a cell surface receptor, which leads to a G protein transmitted signal transduction. The resulting signal is then transmitted and amplified through a mitogen-activated protein (MAP) kinase cascade. A complete cellular response ultimately includes induction or repression of gene transcription, synchronization of the cell cycles (i.e. an arrest in G1 phase), and, finally, the mating of the cells and the fusion of their nuclei [45].

#### Petri net model

**Figure 3:**
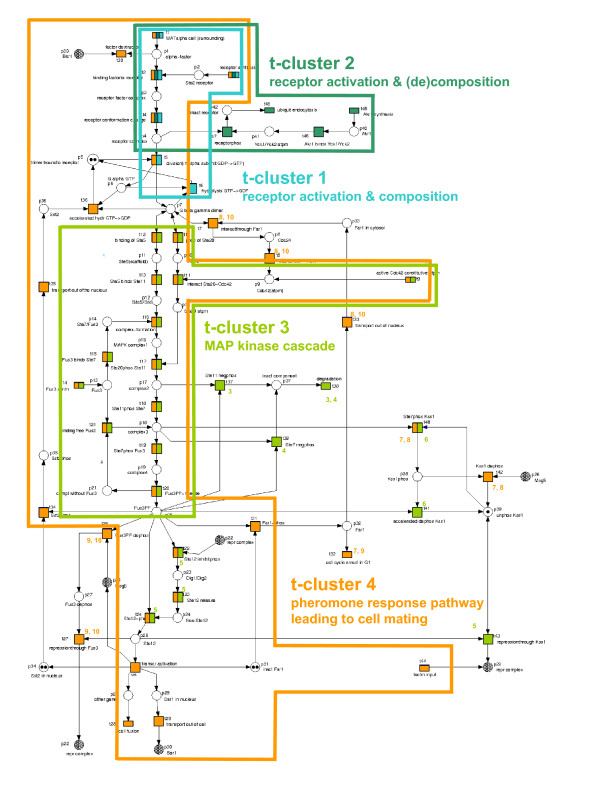
Pheromone response pathway in yeast: the Petri net model. Tables listing the meaning of the places and transitions are given as additional material [see Additional file 3]. The t-invariants are graphically represented using the color code of the respective t-clusters (see Figures 4 and 5). Transitions, which are only included in some of the t-invariants of a given t-cluster, are marked by colored numbers. These numbers correspond to the ID of those t-invariants in which the transition is included. The transitions characterizing a given t-cluster (i.e. those transitions that participate exactly in all t-invariants of the t-cluster) are framed in the color of the respective t-cluster, and the biological meaning of each of these transition sets is denoted.

**Figure 4:**
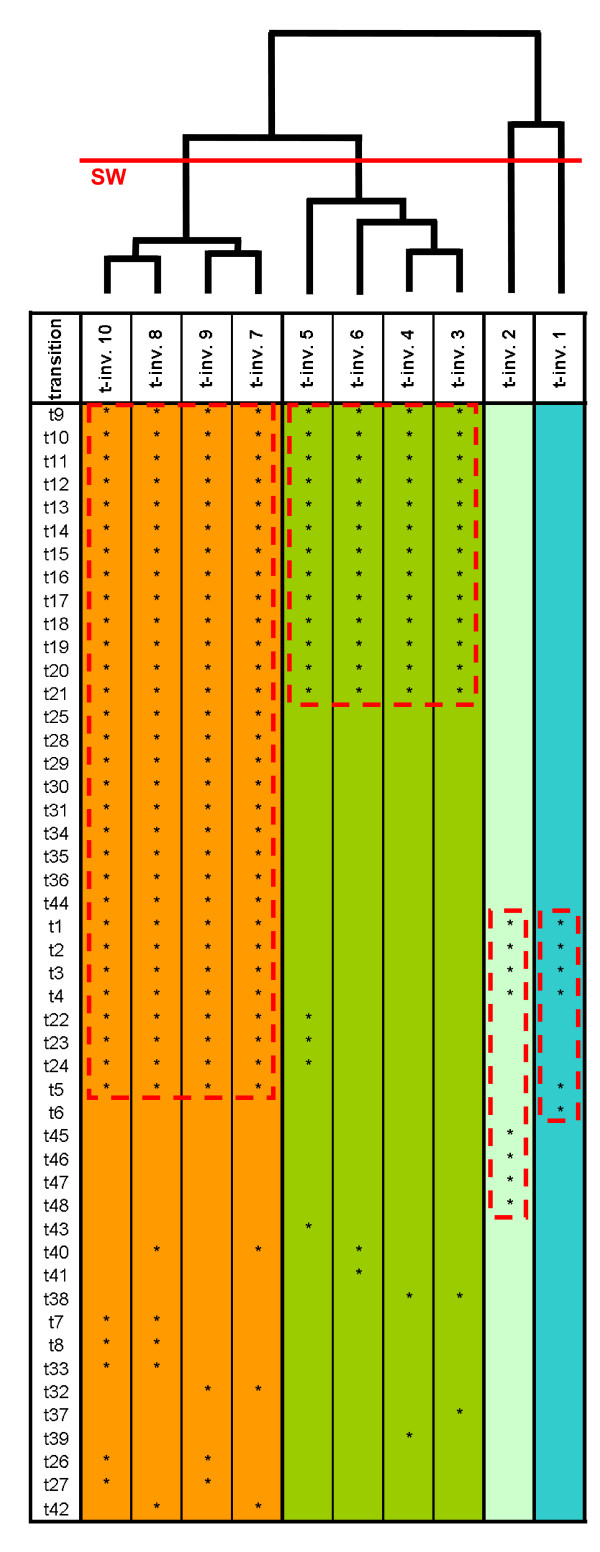
Pheromone response pathway in yeast: the dendrogram of the clustering method UPGMA (distance measure: Tanimoto), with the optimal partition indicated by the cluster validity measure, Silhouette Width (SW). The leaves of the dendrogram correspond to t-invariants. The composition of the t-invariants, based on the transitions, is represented in the subjacent table. The transitions included in a given t-invariant are marked with an asterisk. T-invariants belonging to the same t-cluster, as indicated by the cluster validity measure, Silhouette Width, are of identical color. The transitions characterizing a given t-cluster (i.e. transitions that exactly participate in all t-invariants of the t-cluster) are framed in red.

The Petri net model of the pheromone response pathway, shown in Figure 3, consists of 48 transitions and is covered by 10 t-invariants. The composition of the t-invariants based on the transitions is represented in Figure 4; a table listing the transitions by their name and biological meaning is given as additional material [see Additional file 3]. For further information on the Petri net model the reader is referred to Sackmann *et al*. [10].

#### Clustering results

**Figure 5:**
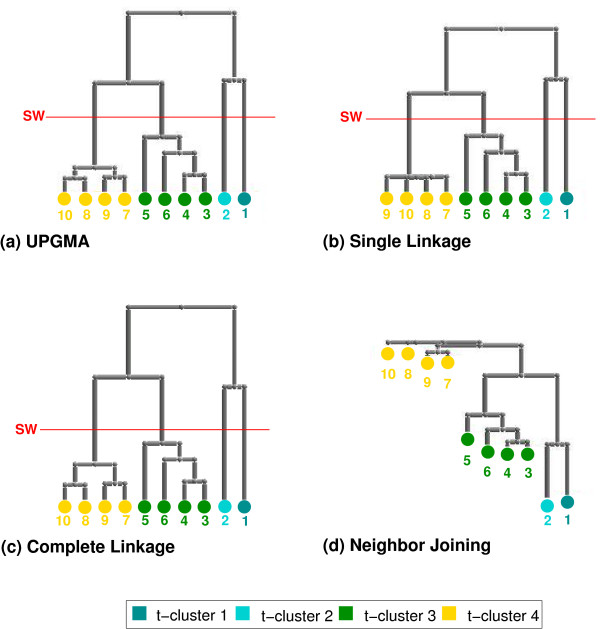
Pheromone response pathway in yeast: the dendrograms of the clustering methods, UPGMA, Single Linkage, Complete Linkage, and Neighbor Joining (distance measure: Tanimoto). The leaves of a given dendrogram correspond to t-invariants characterized by their respective t-invariant number. The optimal partition is indicated by the cluster validity measure, Silhouette Width (SW).

The clustering results of all four methods are shown in Figure 5, with the optimal partition being indicated by the Silhouette Width. Based on this validity measure, the set of the 10 t-invariants is split into four t-clusters by all clustering methods, each cluster containing the same t-invariants across all methods. The composition of the t-invariants belonging to one t-cluster is represented in Figure 4, with the set of transitions characterizing a given t-cluster (i.e. those transitions, which participate exactly in all t-invariants of the t-cluster) being highlighted. A graphical representation as well as a biological interpretation of each of these transition sets is given in Figure 3.

The transition sets shown in Figure 3 define subnets, which can be assigned to a distinct biological meaning. By representing biologically relevant functional units, these subnets can be read as biological modules. With respect to these biological modules (i.e. the common biological features), the t-invariants belonging to one t-cluster are characterized below:

**t-cluster 1: **t-invariant 1

*common biological feature: *receptor activation and composition

The t-invariant 1 includes the synthesis and activation of the pheromone receptor. The activated receptor causes the dissociation of the G-protein, whose subunits reassociate to a trimeric form.

**t-cluster 2: **t-invariant 2

*common biological feature: *receptor activation and (de)composition

The t-invariant 2 includes the synthesis and activation of the pheromone receptor. The phosphorylation of the activated receptor leads to receptor endocytosis.

**t-cluster 3: **t-invariants 3, 4, 5, 6

*common biological feature: *MAP kinase cascade

The t-invariants 3 to 6 include the composition of the MAP kinase cascade complex and the MAP kinase cascade. Differences between the t-invariants originate from actions taking place downstream of the cascade, which either result in the degradation of the MAPK complex (t-invariant 3 (t37, t38)); t-invariant 4 (t38, t39)), or in the repression of a transcription factor (t-invariant 5 (t43), t-invariant 6 (t40, t41)).

**t-cluster 4: **t-invariants 7, 8, 9, 10

*common biological feature: *pheromone response pathway leading to cell mating

The t-invariants 7 to 10 include the complete pheromone response pathway (activation and composition of the pheromone receptor complex, MAP kinase cascade, transcription factor activation, gene transcription, and cell fusion) leading to a mating of the cell. Differences between t-invariants primarily originate from positive and negative feedback regulations, like the repression of transcription factors (t42).

The obtained results show that the t-invariants belonging to one t-cluster are significantly involved in the same biological processes. They are characterized by similar subnets, which correspond to biologically relevant functional modules (see Figure 3). Showing high intra-cluster homogeneity with respect to biological functionality, the computed t-clusters can be considered as biologically meaningful. Thus, all of the applied clustering techniques lead to a meaningful classification of t-invariants.

As shown in Figure 3, the biological modules, characterizing the resulting t-clusters, can be used for decomposing the Petri net model into biologically relevant subnets.

### Gene regulation of Duchenne muscular dystrophy

#### Biological background

Duchenne muscular dystrophy (DMD) is one of the most common inherited human neuromuscular diseases. The disorder is caused by mutations in the dystrophin gene, followed by the absence or functional impairment of the protein. The network downstream of dystrophin, which represents the pathomechanism of DMD, comprises different gene regulatory processes, such as the dystrophin-glycoprotein-complex (DGC) downstream pathway. *DGC *is formed in presence of the protein, dystrophin, which is absent in DMD patients. The functional *DGC *results in a reaction cascade, which finally phosphorylates the transcription factor, *NFATc*. *NFATc *is dephosphorylated and, consequently, activated by calcineurin. In turn, calcineurin is positively regulated by the RAP2B-calcineurin cascade (RAP2B downstream pathway). Activated *NFATc *is able to enter the nucleus and to act as a transcription factor for different genes, such as *MYF5*, *UTRNA*, and *p21*. The cyclin-dependent kinase (CDK) inhibitor, *p21*, is a negative regulator of the G1 to S progression in the cell cycle. Therefore, it plays an important role in cell cycle withdrawal and, consequently, in determining proliferation or differentiation [46].

#### Petri net model

**Figure 6:**
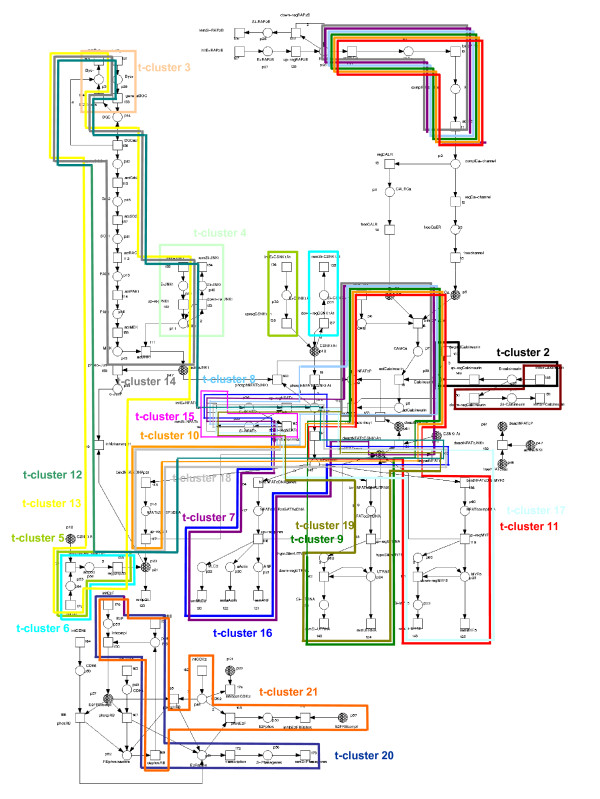
The Petri net model of the gene regulation of Duchenne muscular dystrophy. Tables listing the meaning of the places and transitions are given as additional material [see Additional file 3]. The transitions characterizing a given t-cluster (i.e. the transitions that participate exactly in all t-invariants of the t-cluster) are framed in the color code of the respective t-clusters.

The gene regulatory Petri net model of DMD is shown in Figure 6. The net contains 88 transitions and is covered by 107 t-invariants. A table listing the transitions by their name and biological meaning, is given as additional material [see Additional file 3]. For further information on the Petri net model the reader is referred to Grunwald *et al*. [24].

#### Clustering results

**Figure 7:**
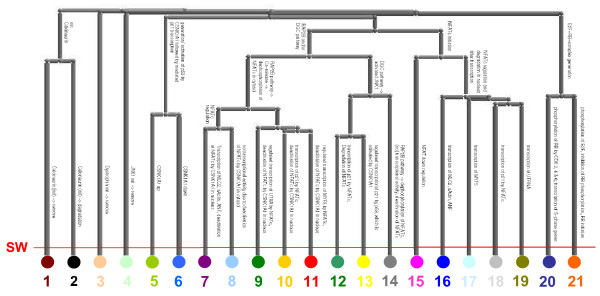
Gene regulation of the Duchenne muscular dystrophy: Dendrogram of the clustering method UPGMA (distance measure: Tanimoto). The leaves of the dendrogram correspond to t-clusters characterized by their respective t-cluster number. Features characterizing a t-cluster are named at the respective arcs of the dendrogram. The optimal partition is indicated by the cluster validity measure Silhouette Width (SW).

Part of the clustering result using UPGMA is shown in Figure 7, with the optimal partition indicated by the Silhouette Width. Based on this validity measure, the set of 107 t-invariants is split into 21 t-clusters. A table depicting the composition of the t-invariants belonging to one t-cluster is given as additional material [see Additional file 4]. The set of transitions characterizing the t-invariants of a given t-cluster are represented in Figure 6. A biological characterization of the t-cluster-specific t-invariants is given below:

**t-cluster 1: **t-invariant 36

initiation, down-regulation, and removal of calcineurin

**t-cluster 2: **t-invariant 5

initiation, up-regulation, and degradation of calcineurin

**t-cluster 3: **t-invariant 34

initiation of dystrophin, followed by generation of *DGC *and simulation of DMD by *DGC *loss

**t-cluster 4: **t-invariant 37

initiation, up-/down-regulation of *JNK1*

**t-cluster 5: **t-invariants 40, 41

initiation and up-regulation of the gene *CSNK1A1*; the protein *CSNK1A1 *activates *p53*, followed by *p21 *transcription

**t-cluster 6: **t-invariants 38, 39

initiation and down-regulation of the gene *CSNK1A1*, the protein *CSNK1A1 *activates *p53*, followed by *p21 *transcription

**t-cluster 7: **t-invariants 100 – 107

regulated RAP2B downstream pathway, including Ca release, which activates regulated *NFATc*, followed by a transcription of *MLC2*, *aActin*, *ANF*, and deactivation of *NFATc *in the nucleus by regulated CSNK1A1

**t-cluster 8: **t-invariants 48, 49, 51, 52, 54, 55, 57, 58

regulated RAP2B downstream pathway, including Ca release, which activates regulated *NFATc*; no transcriptional activity due to deactivation of *NFATc *in the cytosol by regulated *CSNK1A1*

**t-cluster 9: **t-invariants 17, 19, 21, 23, 25, 27, 29, 31

regulated RAP2B downstream pathway, including Ca release, which activates regulated *NFATc*, followed by transcription of *UTRNA*, and deactivation of *NFATc *in the nucleus by regulated *CSNK1A1*

**t-cluster 10: **t-invariants 74 – 77, 80 – 83, 86 – 89, 92 – 95

regulated RAP2B downstream pathway, including Ca release, which activates regulated *NFATc*, followed by *p21 *transcription, and deactivation of *NFATc *in the nucleus by regulated *CSNK1A1*

**t-cluster 11: **t-invariants 16, 18, 20, 22, 24, 26, 28, 30

regulated RAP2B downstream pathway, including Ca release, which activates *NFATc*, followed by transcription of *MYF5*, and deactivation of *NFATc *in the nucleus by regulated *CSNK1A1*

**t-cluster 12: **t-invariant 42

DGC downstream pathway, which activates up-regulated *JNK1*, followed by a *c-JUN *phosphorylation dependent *p21 *inhibition; regulated *NFATc *mediates *p21 *transcription, followed by a degradation of *NFATc *in the nucleus

**t-cluster 13: **t-invariants 32, 33

DGC downstream pathway, which activates regulated *JNK1*, followed by a *c-JUN *phosphorylation dependent *p21 *inhibition; regulation of *CSNK1A1*, which activates *p53*, followed by *p21 *transcription

**t-cluster 14: **t-invariants 8 – 15, 60 – 73, 78, 79, 84, 85, 90, 91, 50, 53, 56, 59, 96, 97, 98, 99

DGC downstream pathway, which activates regulated *JNK1*; regulated RAP2B downstream pathway, which activates regulated *NFATc*, followed by (no) transcription, and deactivation of *NFATc *in the nucleus by *JNK1*

**t-cluster 15: **t-invariant 35

initiation and down-regulation of *NFATc*

**t-cluster 16: **t-invariant 47

regulated *NFATc *mediates transcription of *MLC2*, *aActin*, and *AFN*, followed by degradation of *NFATc *in the nucleus

**t-cluster 17: **t-invariant 7

regulated *NFATc *mediates transcription of *MYF5*, followed by degradation of *NFATc *in the nucleus

**t-cluster 18: **t-invariants 43, 44

regulated *NFATc *mediates *p21 *transcription, followed by degradation of *NFATc *in the nucleus

**t-cluster 19: **t-invariant 6

regulated *NFATc *mediates transcription of *UTRNA*, followed by degradation of *NFATc *in the nucleus

**t-cluster 20: **t-invariants 4, 45, 46

CDK-dependent RB-E2F cell cycle pathway, resulting in transcription of S-phase genes

**t-cluster 21: **t-invariants 1, 2, 3

RB-E2F cell cycle pathway, inhibited by CDK2-phosphorylated *E2F*

In concordance with the results shown in the first case study, the computed t-clusters are biologically meaningful. The t-invariants belonging to one t-cluster are characterized by similar subnetworks, which correspond to biologically relevant functional units (see Figure 6). A distinct separation of t-invariants with respect to biological functionality is shown in the dendrogram given in Figure 7. The t-invariants covering the *RAP2B *downstream pathway (t-clusters 7 – 11, t-cluster 14), the DGC downstream pathway (t-clusters 12 – 14), the *NFATc *initiation (t-clusters 15 – 19), and the *E2F-RB*-complex generation (t-clusters 20, 21) are well-discriminated, and a clear separation concerning the transcriptional targets genes is given. Furthermore, t-invariants representing pathways not covered by any other t-invariant are assigned to singleton clusters (t-cluster 1 – 4), indicating their particular role in the network. Due to the high intra-cluster homogeneity in combination with distinct inter-cluster separation, the application of UPGMA leads to a biologically meaningful classification of t-invariants.

With only minor differences, comparable results are obtained using Complete Linkage and Neighbor Joining. In contrast, the dendrogram based on Single Linkage is characterized by a larger number of singleton t-clusters (i.e. clusters containing only one t-invariant) with only three t-clusters containing more than three t-invariants, thus, resulting in a less distinct discrimination of the t-invariants. The dendrograms, as well as a detailed description of the clustering results, using Single Linkage, Complete Linkage, and Neighbor Joining, are given as additional material [see Additional file 5].

In agreement with the network modularization shown in the first case study, the plotting of the transition sets, characterizing the computed t-clusters, leads to a valuable decomposition of the Petri net model into biologically relevant functional units (see Figure 6).

## Discussion

Our approach aims to facilitate the model validation of biochemical Petri nets. This validation is mainly based on the assignment of a biological meaning to each of the t-invariants. These t-invariants describe possible pathways in the net. Thus, the biological interpretation of t-invariants reflects the system behavior. Model validation of biochemical networks, based on Petri net t-invariants, often becomes unmanageable due to the huge number of t-invariants in large biochemical networks, which can no longer be evaluated manually.

The use of automatic modularization techniques [21,22], which decomposes a complex network into functional modules, facilitates the analysis of complex biological systems and their general behavior. The concept of MCT-sets, introduced by Sackmann *et al*. [10], represents one possible way to decompose t-invariants into disjunctive subnetworks, which can be interpreted as the smallest biological building blocks. Another possibility is the clustering of t-invariants, as described in this paper, which generally results in overlapping subnetworks. This method can be applied to large sets of t-invariants, with the user having the ability to influence the complexity of the evaluation by choosing a respective number of t-clusters for interpretation.

Data mining techniques, such as cluster analysis, enable the analysis of large sets of data, due to the identification of relatively homogeneous subsets, resulting in a structured and reduced data representation. To investigate the classification of biochemical Petri net t-invariants based on cluster analysis, we have applied different distance measures and clustering techniques to various sets of t-invariants of biochemical Petri net models, using as examples, a small signal transduction pathway and a larger gene regulatory pathway. To identify the optimal number of t-clusters to consider for interpretation, different cluster validity measures (Silhouette Width, Dunn-, Davies-Bouldin-, and C-index) have been evaluated in preliminary investigations, with the Silhouette Width offering the best results with respect to the percentage of correct predictions. The clustering techniques, UPGMA, Single Linkage, Complete Linkage, and Neighbor Joining, have been tested in combination with different distance measures (Tanimoto, Simple Matching, Sum of Difference). With respect to the biological interpretability, the best results are obtained using the Tanimoto distance measure. In combination with the Tanimoto coefficient, our results suggest that the clustering techniques, UPGMA, Complete Linkage, and Neighbor Joining are suitable for the clustering of t-invariants. All of the clustering results based on these methods correspond to a biologically meaningful classification of t-invariants in the example pathways. The computed t-clusters comprise t-invariants, which are significantly involved in the same biological processes. While t-invariants characterized by similar subpathways are grouped together, t-invariants, which correspond to subpathways not covered by any other t-invariant, are assigned to singleton clusters (e.g. Figure 7). These "outliers" have to be checked carefully with respect to biological functionality, as they might represent a modeling error. In contrast to the clustering results obtained by UPGMA, Complete Linkage, and Neighbor Joining, a functionally indistinct classification is obtained using the Single Linkage method (see e.g. the second case study). Being subject to a chaining effect [47], the Single Linkage algorithm has the tendency to produce straggling or elongated clusters and is less eligible for the classification of t-invariants. When dealing with large numbers of t-invariants, Neighbor Joining is outperformed by UPGMA and Complete Linkage due to its run-time complexity. On that account, our results suggest that UPGMA and Complete Linkage are the most appropriate methods for a biological classification of t-invariants.

The set of transitions that characterizes a given t-cluster of t-invariants can be interpreted as a biological module. Similar to the MCT-sets, these modules are automatically generated based on structural network properties only, and can be assigned to a distinct biological meaning. Whereas MCT-sets describe disjunctive subnetworks, t-clusters can produce overlapping subnetworks. These overlapping transitions can be interpreted as a set of compounds (or enzymes for metabolic networks) that are active in more than one functional module. By decomposing a given network into biochemical subnetworks, our approach demonstrates a biologically meaningful modularization technique based on the clustering of t-invariants.

## Conclusion

This paper describes a new general approach for the decomposition of large biochemical networks into functional modules. This decomposition is based on Petri net t-invariants, but can also be applied to elementary modes. We have used different clustering techniques in combination with different distance measures and cluster validity measures to classify t-invariants. The obtained results suggest that UPGMA and Complete Linkage, in combination with the Tanimoto distance measure, and the cluster validity measure, Silhouette Width, are suitable for classifying t-invariants into t-clusters that have a distinct biological meaning. T-invariants of a given t-cluster are significantly involved in the same biological processes. They are characterized by similar subnets, which correspond to biologically relevant functional units. Our approach leads to a biologically meaningful data reduction and structuring of the network. Thus, large sets of t-invariants can be evaluated and interpreted, allowing for model validation of biochemical systems.

## Availability and requirements

The Petri nets have been edited with the graphical Petri net editor and animator *Snoopy *[48]. The analyses have been done using the Integrated Net Analyzer *INA *[49]. Cluster analysis has been performed using *PInA *[50], and the constructed dendrograms have been visualized with *Wilmascope *[51,52]. All programs are freely available.

## Authors' contributions

IK and FS designed the study. EGB and IK drafted the manuscript. MH, IK, and FS revised the manuscript. EGB and KW implemented the software. SG, BJ, AnS, and AsS provided the models and participated in the biological analysis and interpretation of the results. All authors read and approved the final manuscript.

## Supplementary Material

Additional File 1 Distance measures. In the PDF file, DistanceMeasures.pdf, a mathematical definition and evaluation of the distance measures tested in preliminary investigations is given. Click here for file

Additional File 2 Cluster validity measures. In the PDF file, ClusterValidityMeasures.pdf, a mathematical definition and evaluation of the cluster validity measures tested in preliminary investigations is given. Click here for file

Additional File 3 Petri net models. In the ZIP file, PetriNetModels.zip, the Petri net models of the two case studies are provided. In addition, tables, listing the transitions and places of the models by their name and biological meaning, are given. Click here for file

Additional File 4 T-invariants of the Petri net model of DMD. Description: In the PDF file, DMDTinvariants.pdf, a table, depicting the composition of the t-invariants of the Petri net model of DMD based on the transitions, is provided. Click here for file

Additional File 5 Clustering results of the Petri net model of DMD. In the ZIP file, DMDClusteringResults.zip, the clustering results of the Petri net model of DMD, using Single Linkage, Complete Linkage, and Neighbor Joining, are provided. For each method, the constructed dendrogram as well as a detailed description of the clustering result is given. Click here for file
