# Supplementary material for: Modularization of biochemical networks based on classification of Petri net t-invariants
Source: BMC Bioinformatics. 2008 Feb 8;9:90. doi: 10.1186/1471-2105-9-90 (PMC2277402; doi:10.1186/1471-2105-9-90)
Supplement: Additional File 1 — Distance measures. In the PDF file, DistanceMeasures.pdf, a mathematical definition and evaluation of the distance measures tested in preliminary investigations is given. [file 1471-2105-9-90-S1.pdf]

Distance measures

Several distance measures (*Tanimoto*, *Simple Matching*, and *Sum of Differences* (SOD)) have been tested in preliminary investigations. In the following the definitions of the applied distance measures are given and the obtained results are discussed. For a more formal and detailed description of the distance measures the reader is referred to the respective literature given at the end of this material.

Simple Matching [Backhaus *et al.*, 2000]
Considering the support vectors t_i and t_j describing two t-invariants, the Simple Matching coefficient is defined as:

$$s(t_i, t_j) = s_{ij} = \frac{a + e}{a + b + c + e},$$

where a is the number of features (i.e. transitions) present in both t-invariants, b is the number of features only present in t_i, c is the number of features only present in t_j, and e is the number of features not present in both t-invariants. To measure the distance based on the Simple Matching coefficient, s_{ij} has to be transformed into a distance d_{ij} [Steinhausen, 1977] by

$$d_{ij} = 1 - s_{ij}$$

Sum of differences (SOD) [Grunwald *et al.*, 2007]
Unlike Tanimoto and Simple Matching, the distance measure SOD is applied to the Parikh vector. Considering the Parikh vectors t_i and t_j describing two t-invarints, the distance measure SOD is defined as:

$$d(t_i, t_j) = d_{ij} = \frac{a}{b},$$

where a is the sum of the absolute differences between each component of the Parikh vectors t_i and t_j, and b is the number of transitions occurring in at least one of the two t-invariants, thereby serving the purpose of normalization

Based on an external cluster validation (i.e. the evaluation of clustering results based on the knowledge of the correct classification of objects) the distance measures were validated and compared on the basis of various sets of t-invariants of different types of Petri nets (i.e. metabolic, gene regulatory and signal transduction nets). With respect to the biological interpretability, best results were obtained using the distance measure Tanimoto. The distance measure Simple Matching is a binary feature vector-based measure commonly used in cluster analysis. Unlike Tanimoto, Simple Matching additionally takes into account the non-existence of features in both objects. The distance measure is useful when both positive and negative features (i.e. existing and non-existing features, respectively) carry equal information. Since the aim of the approach is a classification of t-invariants into functionally distinct t-clusters, i.e. with respect to their biological meaning, it is a useful procedure to link the similarity between invariants to those features being present in both rather than those ones being absent in both invariants. Thus, a Simple Matching based cluster assignment predominantly based on the non-existing features (i.e. on those transitions not included in the invariants) leads to a less distinct classification, as shown in some of the used cases. Unlike Tanimoto and Simple Matching, the distance measure SOD is based on the Parikh vector of the t-invariants, thus taking into account the firing frequency of the respective transitions. Since we analyze and cluster the invariant set of qualitative Petri nets, this feature is of minor importance as the biological function of a t-invariant is encoded by the presence of its respective transitions (i.e. the support vector) rather than the firing frequency of the transitions. The obtained clustering results confirm this choice.

References

Backhaus *et al.*, 2000. Backhaus, K., Erichson, B., Plinke, W., Weiber, R. (Hg.) (2000). Multivariate analysis methods. An application-oriented introduction (in German). Springer, Berlin.

Grunwald *et al.*, 2007. Grunwald, S. and Speer, A. and Ackermann, J. and Koch, I. (2007) Petri net modelling of gene regulation of the Duchenne muscular dystrophy. *BioSystems*, **92**(2),*in press*.

Steinhausen, 1977. Steinhausen, D. and Langer, K. (Hg.) (1977). Cluster analysis. An Introduction to methods for automatic classification (in German). de Gruyter, Berlin.
