# Supplementary material for: Modularization of biochemical networks based on classification of Petri net t-invariants
Source: BMC Bioinformatics. 2008 Feb 8;9:90. doi: 10.1186/1471-2105-9-90 (PMC2277402; doi:10.1186/1471-2105-9-90)
Supplement: Additional File 2 — Cluster validity measures. In the PDF file, ClusterValidityMeasures.pdf, a mathematical definition and evaluation of the cluster validity measures tested in preliminary investigations is given. [file 1471-2105-9-90-S2.pdf]

Cluster validity measures

Different validity measures (Silhouette Width, Dunn-, Davies-Bouldin- and C-index) have been tested in preliminary investigations. In the following the definitions of the applied validity measures are given and the obtained results are discussed. For a more formal and detailed description of the validity measures the reader is referred to the respective literature given at the end of this material..

Dunn index [Dunn, 1974]
The Dunn index defines the ratio between the minimal intracluster distance to maximal intercluster distance. The index is given by:

$$D = \frac{d_{min}}{d_{max}},$$

where d_{min} denote the smallest distance between two objects ¿from different clusters, and d_{max} the largest distance of two objects from the same cluster. The Dunn index is limited to the interval $[0, \infty]$ and should be maximized.

Davies-Bouldin index [Davies & Bouldin, 1979]
This index, DB, is defined as:

$$DB = \frac{1}{n} \sum_{i=1, i \neq j}^{n} max \left(\frac{\sigma_i + \sigma_j}{d(c_i, c_j)} \right),$$

where n is the number of clusters, σ_i is the average distance of all patterns in cluster i to their cluster center c_i, σ_j is the average distance of all patterns in cluster j to their cluster center c_j, and $d(c_i, c_j)$ is the distance of cluster centers c_i and c_j. Small values of DB correspond to clusters that are compact, and whose centers are far away from each other. Consequently, the number of clusters that minimizes DB is taken as the optimal number of clusters.

C-index [Hubert, 1976]
The C-index is defined as:

$$C = \frac{S - S_{min}}{S_{max} - S_{min}},$$

where S is the sum of distances over all pairs of objects form the same cluster, n is the number of those pairs and S_{min} is the sum of the n smallest distances if all pairs of objects are considered. Likewise S_{max} is the sum of the n largest distances out of all pairs. The C-index is limited to the interval [0, 1] and should be minimized.

Based on an external cluster validation the validity measures were evaluated and compared on the basis of various sets of t-invariants of different types of Petri nets (i.e. metabolic, gene regulatory and signal transduction nets). With respect to the percentage of correct predictions best results were obtained using the Silhouette Width (75%) and the C-index (75%), followed by the Dunn-index (50%) and DaviesBouldin index (8%). Although offering good results, the C-index is hampered by the fact of showing optimal index values for different numbers of clusters, thus impeding a robust automatic determination of the optimal number of clusters. Given the noisy nature of biological data, robust measures like the Silhouette Width are preferable to noise-sensitive measures like the Dunn index, which is instable against outliers due to the consideration of only two distances. The Davies-Bouldin index requires the computation of the cluster center, which cannot achieved by average determination when dealing with binary data. An inappropriate choice of method for cluster center determination might have been one of the reasons for the insufficient clustering results obtained by this distance measure.

References

Davies & Bouldin, 1979. Davies, D.L., Bouldin, D.W., (2000) A cluster separation measure. *IEEE Trans. Pattern Anal. Machine Intell.*, **1(4)**, 224-227.

Dunn, 1974. Dunn, J. (1974) Well separated clusters and optimal fuzzy partitions. *Journal of Cybernetics* , **4**, 95-104.

Hubert, 1976. Hubert, L. and Schultz, J. (1976) Quadratic assignment as a general data-analysis strategy. *British Journal of Mathematical and Statistical Psychology*, **29**, 190-241.

Rousseeuw, 1987. Rousseeuw, P.J., (1987) Silhouettes: a graphical aid to the interpretation and validation of cluster analysis. *Journal of Computational and Applied Mathematics*, **20**, 53-65.
