# Supplementary material for: Modularization of biochemical networks based on classification of Petri net t-invariants
Source: BMC Bioinformatics. 2008 Feb 8;9:90. doi: 10.1186/1471-2105-9-90 (PMC2277402; doi:10.1186/1471-2105-9-90)
Supplement: Additional File 3 — Petri net models. In the ZIP file, PetriNetModels.zip, the Petri net models of the two case studies are provided. In addition, tables, listing the transitions and places of the models by their name and biological meaning, are given. [file 1471-2105-9-90-S3.zip › PetriNetModels/DMD_ModelDescription.pdf]

Gene regulation of the Duchenne muscular dystrophy

Table 1 - The transitions of the model.

The 88 transitions of the gene regulatory Petri net model of DMD, each transition listed with its name, its ID and the biological event represented by this node.

ID	Transition name	Biological event
t0	*bind_PIP2*	complex formation of PIP2 and PLCe
t1	*act_IP3*	activation of IP3 by *p1*
t2	*reg_Ca-channel*	Ca-channel mediates Ca-release depending on concentration gradient between endoplasmatic reticulum (ER) and cytosol
t3	*reg_CALR*	regulation of Calreticulin, which binds to calcium
t4	*free_CALR*	calcium release regulated by calreticulin
t5	*free_channel*	calcium release regulated by concentration gradient
t6	*bind_CAM_Ca*	calmodulin binds to calcium
t7	*gen_CAM*	generation of calmodulin
t8	*act_Calcineurin*	activation of calcineurin by calmodulin
t9	*phos_NFATc_CSNK1A1*	phosphorylation of *NFATc* by *CSNK1A1*
t10	*dephosph*	dephosphorylation of *NFATc* by active calcineurin
t11	*act_JNK1*	activation of *JNK1* by *MEK*
t12	*act_RAC1*	activation of *RAC1* by *SOS1*
t13	*act_Grb2*	activation of *Grb2* by active *DGC*
t14	*act_PAK1*	activation of *PAK1* by *RAC1*
t15	*bind_NFATc_DNA_p21*	*NFATc* activated by calcineurin binds to DNA to transcribe *p21*
t16	*up_reg_genes*	transcription regulation of subsequent genes
t17	*up_reg_p21*	transcription regulation of *p21*
t18	*up_reg_UTRNA*	transcription regulation of *UTRNA*
t19	*up_reg_MYF5*	transcription regulation of *MYF5*
t20	*rem_MLC2*	removal of *MLC2* out of the system
t21	*rem_ANF*	removal of *ANF* out of the system
t22	*rem_aActin*	removal of *aActin* out of the system
t23	*rem_p21*	removal of *p21* out of the system
p24	*rem_UTRNA*	removal of *UTRNA* out of the system
t25	*rem_MYF5*	removal of *MYF5* out of the system
t26	*rem_Si_RAP2B*	removal of silencing *RAP2B* out of the system
t27	*init_E_RAP2B*	initiation of enhancer of *RAB2B*
t28	*down_reg_RA2B*	down-regulation of *RAP2B*
t29	*up_reg_RAP2B*	up-regulation of *RAP2B*
t30	*act_PLCe*	activation PLCe by *RAP2B*
t31	*init_Dys*	initiation of dystrophin
t32	*rem_Dys*	removal of dystrophin out of the system
t33	*generat_DGC*	generating the *DGC*
t34	*DGC_loss*	loss of the *DGC* due to missing dystrophin
t35	*rem_Si_CSNK1A1*	removal of silencing *CSNK1A1* out of the system
t36	*init_E_CSNK1A1*	initiation of enhancer of *CSNK1A1*
t37	*down_reg_CSNK1A1*	down-regulation of *CSNK1A1*
t38	*up_reg_CSNK1A1*	up-regulation of *CSNK1A1*
t39	*inhib_transcr_p21*	transcription inhibition of *p21*
t40	*rem_Si_UTRNA*	removal of silencing *UTRNA* out of the system
t41	*down_reg_MYF5*	down-regulation of *MYF5*
t42	*down_reg_UTRNA*	down-regulation of *UTRNA*

t43	rem_Si_MYF5	removal of silencing *MYF5* out of the system
t44	rem_Si_NFATc	removal of silencing *NFATc* out of the system
t45	$down\_reg\_NFATc$	down-regulation of *NFATc*
t46	up_reg_NFATc	up-regulation of *NFATc*
t47	$init\_E\_NFATc$	initiation of enhancer of *NFATc*
t48	$init\_E\_Calcineurin$	initiation of enhancer of calcineurin
t49	$up\_reg\_Calcineurin$	up-regulation of calcineurin
t50	$down\_reg\_Calcineurin$	down-regulation of calcineurin
t51	$rem\_Si\_Calcineurin$	removal of silenced calcineurin out of the system
t52	up_reg_JNK1	up-regulation of *JNK1*
t53	$down\_reg\_JNK1$	down-regulation of *JNK1*
t54	rem_Si_JNK1	removal of silenced *JNK1* out of the system
t55	$init\_E\_JNK1$	initiation of enhancer of *JNK1*
t56	DGC_act	activating the *DGC*
t57	act_SOS1	activation of *SOS1* by active *Grb2*
t58	act_MEK	activation of *MEK* by active *PAK1*
t59	$phos\_c\text{-}Jun$	phosphorylation of *c_Jun* by active *JNK1*
t60	$phos\_NFATc\_JNK1$	phosphorylation of *NFATc* by active *JNK1*
t61	$Transfactor\_in\_nuc$	transcription factor *NFATc* migrates into the nucleus
t62	$init\_CDK2$	initiation of CDK2
t63	$init\_CDK4$	initiation of CDK4
t64	$init\_CDK6$	initiation of CDK6
t65	$phosph\_RB$	phosphorylation of *RB* by CDK2
t66	$phos\_RB$	phosphorylation of *RB* by CDK6
t67	$phosp\_RB$	phosphorylation of *RB* by CDK4
t68	$phos\_E2F$	phosphorylation of *E2F* by CDK2
t69	$dephos\_RB$	dephosphorylation of *RB*
t70	$init\_compl$	initiation of complex of *E2F* and *RB*
t71	$init\_RB$	initiation of *RB*
t72	$transcription$	transcription of S-phase genes mediated by *E2F*
t73	$rem\_S-Phase\_genes$	removal of S-phase genes out of the system
t74	$inhib\_p21\_CDK2$	inhibition of CDK2 by *p21*
t75	$inhib\_E2F\_RBphos$	inhibition of *RB* phosphorylation by *E2F*
t76	$init\_E2F$	initiation of *E2F*
t77	$phos\_p53\_CSNK1A1$	phosphorylation of *p53* by *CSNK1A1*
t78	reg_p21_p53	transcription regulation of *p21* by *p53*
t79	$init\_p53$	initiation of *p53*
t80	$degrad\_NFATc$	degradation of *NFATc* by protein catabolism of the cell
t81	$deact\_NFATc\_CSNK1A1$	deactivation of *NFATc* by *CSNK1A1*
t82	$deact\_NFATc\_JNK1$	deactivation of *NFATc* by *JNK1*
t83	$Transfactor\_in\_cyt$	transcription factor into cytosol
t84	$bind\_NFATc\_DNA\_genes$	*NFATc* binds to DNA of genes
t85	$bind\_NFATc\_DNA\_UTRNA$	*NFATc* binds to DNA of *UTRNA*
t86	$bind\_NFATc\_DNA\_MYF5$	*NFATc* binds to DNA of *MYF5*
t87	$degrad\_Calcineurin$	degradation of calcineurin

Table 2 - The places of the model.
The 64 places of the gene regulatory Petri net model of DMD, each transition listed with its name, its ID and the biological species represented by this node.

ID	Transition name	Biological species
p0	*PLCe*	Phospholipase C
p1	*compPIP2_PLCe*	Complex of PIP2 and PLCe
p2	*compl_Ca-channel*	Complex of IP3 FKBP12 bound to Ca channel
p3	*free_Ca_ER*	Complex of IP3 FKBP12 bound
p4	*CALR_Ca*	Calreticulin bound to calcium
p5	*Ca*	Free calcium
p6	*CAM*	Calmodulin
p7	*CAM_Ca*	Calcium activated calmodulin
p8	*act_Calcineurin*	Calmodulin activated calcineurin
p9	*NFATc_P*	Phosphorylated *NFATc*
p10	*NFATc*	Nuclear factor of activated T-cells
p11	*JNK1*	c–Jun N-terminal kinase 1
p12	*CSNK1A1*	Casein kinase 1 alpha 1
p13	*RAC1*	Ras-related C3 botulinum toxin substrate 1
p14	*DGC*	Dystrophin glycoprotein complex
p15	*Grb2*	Growth factor receptor bound protein 2
p16	*PAK1*	RAC1-activated kinase 1
p17	*NFATc_MEF2c_GATA4_DNA*	Transcription factor complex of *NFATc*, *MEF2c*, *GATA4* bound to DNA
p18	*NFATc_SP1_SP3_DNA*	*NFATc* in complex with *SP1* and *SP3* bound to DNA
p19	*NFATc_com_DNA*	*NFATc* in complex with other Transcription factors bound to DNA
p20	*aActin*	Alpha actin
p21	*ANF*	Atrial natriuretic factor precursor
p22	*MLC2*	Myosin light chain 2
p23	*p21*	CDKN1-Cyclin-dependent kinase Inhibitor 1
p24	*UTRNA*	Utrophin A
p25	*MYF5*	Myogenic factor 5
p26	$Si - RAP2B$	Silencer of *RAP2B*
p27	$E + RAP2B$	Enhancer of *RAP2B*
p28	*RAP2B*	Ras related protein 2B
p29	*Dys+*	Dystrophin positive
p30	*Dys-*	Dystrophin negative
p31	*Si-CSNK1A1*	Silencer of *CSNK1A1*
p32	*E+CSNK1A1*	Enhancer of *CSNK1A1*
p33	*Si-MYF5*	Silencer of *MYF5*
p34	*Si-UTRNA*	Silencer of *UTRNA*
p35	*Si-NFATc*	Silencer of *NFATc*
p36	*E+NFATc*	Enhancer of *NFATc*
p37	*E+calcineurin*	Enhancer of calcineurin
p38	*Si-Calcineurin*	Silencer of calcineurin
p39	*E+JNK1*	Enhancer of *JNK1*
p40	*Si-JNK1*	Silencer of *JNK1*
p41	*SOS1*	Guanine nucleotide exchange factor
p42	*active_DGC*	Active *DGC*
p43	*MEK*	Mitogen activated protein kinase 1

p44	$c\text{-}Jun\_P$	Phosphorylated *c-Jun*
p45	$NFATc\_nuc$	Activated *NFATc* in nucleus
p46	$free\_NFATc\_nuc$	Free *NFATc* in nucleus
p47	$active\_JNK1$	Activated *JNK1*
p48	$CDK2$	Cyclin-dependent kinase 2
p49	$CDK4$	Cyclin-dependent kinase 4
p50	$CDK6$	Cyclin-dependent kinase 6
p51	$E2F\_active$	Activated *E2F*
p52	$RB\_phos\_inactive$	*RB* inactivated by phosphorylation
p53	$E2F\_phos$	Phosphorylated *E2F*
p54	RB	Retinoblastoma protein
p55	$E2F$	Transcription factor *E2F*
p56	$S\text{-}phase\_genes$	Genes mediating the S-phase of the cell cycle
p57	$E2F\_RB\_compl$	Complex composed of *E2F* and *RB*
p58	act_p53	Activated tumour protein *p53*
p59	$Calcineurin$	Calcineurin - protein phosphatase 2B
p60	$NFATc\_comp\_DNA$	*NFATc* in complex with other
p61	$deact\_NFATc\_P$	Deactivated *NFATc*
p62	hypo_Silen_UTRNA	Hypothetical silencer of *UTRNA*
p63	hypo_Silen_MYF5	Hypothetical silencer of *MYF5*
