# Supplementary material for: Modularization of biochemical networks based on classification of Petri net t-invariants
Source: BMC Bioinformatics. 2008 Feb 8;9:90. doi: 10.1186/1471-2105-9-90 (PMC2277402; doi:10.1186/1471-2105-9-90)
Supplement: Additional File 3 — Petri net models. In the ZIP file, PetriNetModels.zip, the Petri net models of the two case studies are provided. In addition, tables, listing the transitions and places of the models by their name and biological meaning, are given. [file 1471-2105-9-90-S3.zip › PetriNetModels/PheromoneModelDescription.pdf]

## Pheromone Response pathway in yeast

### Table 1 - The transitions of the model.

The 48 transitions of the Petri net model, each transition listed with its name, its ID and the biological event represented by this node.

| ID | Transition name | Biological event |
|---|---|---|
| t1 | MATalpha_cell(surroundings) | a near MAT$\alpha$ cell secretes its mating pheromone |
| t2 | binding_factor_to_receptor | the $\alpha$-factor binds to the Ste2 receptor |
| t3 | receptor_synthesis | synthesis of the cell surface receptor Sst2 |
| t4 | receptor_conformation_change | conformation change of the receptor |
| t5 | division(in_alpha_subunit:GDP− >GTP) | dissociation of the G$\alpha$ subunit of the G-protein |
| t6 | hydrolysis_GTP− >GDP | hydrolysis reassociates G$\alpha$ with G$\beta\gamma$ |
| t7 | interact_through_Far1 | G$\beta\gamma$ interacts Far1 transmitted with Cdc24 |
| t8 | Cdc42:GDP− >GTP | Cdc24 supported activation of Cdc42 |
| t9 | active_Cdc42_constitutive_at_pm | constitutive active Cdc42 attending the processes |
| t10 | binding_to_Ste20 | G$\beta\gamma$ binds Ste20 |
| t11 | Ste20_activated | Cdc42 at plasma membrane activates Ste20 |
| t12 | binding_to_Ste5 | G$\beta\gamma$ binds Ste5 |
| t13 | Ste5_binds_Ste11 | Ste5 binds Ste11 |
| t14 | Fus3_synth | synthesis of kinase Fus3 |
| t15 | Fus3_binds_Ste7 | Ste7 binds Fus3 |
| t16 | complex-formation | Ste5/Ste11 binds Ste7/Fus3 |
| t17 | Ste20_phos_Ste11 | phosphorylation of Ste11 by Ste20 |
| t18 | Ste11_phos_Ste7 | phosphorylation of Ste7 by Ste11 |
| t19 | Ste7_phos_Fus3 | phosphorylation of Fus3 by Ste7 |
| t20 | Fus3PP-release | release of activated Fus3 out of the MAPK complex |
| t21 | binding_free_Fus3 | remaining MAPK complex binds Fus3 |
| t22 | Ste12_inhibit_phos | phosphorylation of Ste12 inhibitors Dig1/Dig2 by Fus3PP |
| t23 | Ste12-release | release of Ste12 out of the repression complex |
| t24 | Ste12_phos | phosphorylation of Ste12 by Fus3PP |
| t25 | transcr_activation | transcription activation of pheromone regulated genes |
| t26 | Fus3PP_dephos | dephosphorylation of Fus3PP by Msg5 |
| t27 | repression_through_Fus3 | Ste12 repression through inactive Fus3 and Dig1/Dig2 |
| t28 | cell_fusion | processes leading to the fusion of the two haploid cells |
| t29 | transport_out_of_cell | Bar1 transport into the cell environment |
| t30 | factor_destruction | Bar1 transmitted destruction of the $\alpha$-factor |
| t31 | Far1_phos | phosphorylation of Far1 by Fus3PP |
| t32 | cell_cycle_arrest_in_G1 | Far1 caused arrest in the cell cycle phase G1 |
| t33 | transport_out_of_nucleus | Far1 transport out of the nucleus |
| t34 | Sst2_phos | phosphorylation of Sst2 by Fus3PP |
| t35 | transport_out_of_nucleus | Sst2 transport out of the nucleus |
| t36 | accelerated_hydr_GTP− >GDP | accelerated hydrolysis reassociates the G-protein |
| t37 | Ste11_neg_phos | Fus3PP labels the MAPK complex at Ste11 for degradation |
| t38 | degradation | degradation of the MAPK complex |
| t39 | Ste7_neg_phos | Fus3PP labels the MAPK complex at Ste7 for degradation |
| t40 | Ste7_phos_Kss1 | phosphorylation of Kss1 by Ste7 |
| t41 | accelerated_dephos_Kss1 | deactivation of phosphorylated Kss1 by Fus3PP |
| t42 | Kss1_dephos | dephosphorylation of phosphorylated Kss1 by Msg5 |
| t43 | repression_through_Kss1 | Ste12 repression through inactive Kss1 and Dig1/Dig2 |
| t44 | techn_input | technical: the repressed Ste12 complex assumed to be present |

| | | |
|---|---|---|
| t45 | Akr1_synthesis | synthesis of Akr1 |
| t46 | Akr1_binds_Yck1/Yck2 | Akr1 binds Yck1/Yck2 |
| t47 | receptor_phos | labelling of Ste2 for degradation |
| t48 | ubiquit_endocytosis | ubiquitination and endocytosis of the receptor |

**Table 2 - The places of the model.**

The 42 places of the model, each listed with its name, its ID and the biological species represented by this node.

| ID | Place name | Biological species |
|---|---|---|
| p1 | alpha-factor | pheromone released by an MAT$\alpha$ cell in the surroundings |
| p2 | Ste2_receptor | mating pheromone receptor of the modelled MAT**a** cell |
| p3 | receptor_factor_complex | complex consisting of the $\alpha$-factor and the Ste2 receptor |
| p4 | receptor_complex | the above named complex is activated by a conformation change |
| p5 | trimer_bound_to_receptor | heterotrimeric G protein, which is coupled to the Ste2 receptor |
| p6 | G_alpha_GTP | dissociated G$\alpha$ subunit (exchange of GDP to GTP in this monomer) |
| p7 | G_beta_gamma_dimer | G-protein G$\beta\gamma$ subunits in a dimeric form |
| p8 | Cdc24 | Cdc24, i.e., guanine nucleotide exchange factor of Cdc42 |
| p9 | Cdc42(at_pm) | Cdc42 located at the plasma membrane |
| p10 | Ste20 | protein kinase Ste20 |
| p11 | Ste5(scaffold) | Ste5, acting as a scaffold protein |
| p12 | Ste5/Ste11 | protein complex consisting of Ste5 and Ste11 |
| p13 | Fus3 | MAP kinase Fus3 |
| p14 | Ste7/Fus3 | protein complex consisting of Ste7 and Fus3 |
| p15 | MAPK_complex1 | MAPK complex consisting of Ste5, Ste11, Ste7 and Fus3 |
| p16 | Ste20_at_pm | Ste20 located at the plasma membrane, i.e., near the MAPK complex |
| p17 | complex2 | as complex1, but Ste11 is activated additionally |
| p18 | complex3 | as complex2, but Ste7 is activated additionally |
| p19 | complex4 | as complex3, but Fus3 is activated additionally |
| p20 | Fus3PP | dissociated Fus3 in the activated form |
| p21 | compl_without_Fus3 | as complex4, but without Fus3 |
| p22 | repr_complex | complex containing Ste12 repressed by Fus3 or Kss1 and Dig1/Dig2 |
| p23 | Dig1/Dig2 | Ste12 inhibitors, i.e., cofactors for the repression |
| p24 | free_Ste12 | Ste12 released out of the repression complex |
| p25 | Ste12 | activated transcription factor Ste12 |
| p26 | Msg5 | phosphatase Msg5 being able to deactivate Fus3 or Kss1 |
| p27 | Fus3_dephos | deactivated Fus3 |
| p28 | other_genes | pheromone regulated genes encoding mating related cell responses |
| p29 | Bar1_in_nucleus | synthesised protease Bar1 located in the nucleus |
| p30 | Bar1 | Bar1 secreted in the cell environment |
| p31 | inact_Far1 | synthesised Far1 located in the nucleus in an inactive form |
| p32 | Far1 | Far1 activated by phosphorylation |
| p33 | Far1_in_cytosol | active Far1 located in the cytosol |
| p34 | Sst2_in_nucleus | synthesised Sst2 located in the nucleus in an inactive form |
| p35 | phos_Sst2 | Sst2 activated by phosphorylation |
| p36 | Sst2 | active Sst2 located in the cytosol |
| p37 | inact_component | complex labelled for degradation by phosphorylation |
| p38 | phos_Kss1 | MAP kinase Kss1 activated by phosphorylation |
| p39 | unphos_Kss1 | inactive Kss1 |
| p40 | Akr1 | protein Akr1 located at plasma membrane |
| p41 | Yck1/Yck2_at_pm | kinases Yck1/Yck2 being able to label the Ste2 for degradation |
| p42 | inact_receptor | receptor labelled for ubiquitination and endocytosis |
