# Supplementary material for: Modularization of biochemical networks based on classification of Petri net t-invariants
Source: BMC Bioinformatics. 2008 Feb 8;9:90. doi: 10.1186/1471-2105-9-90 (PMC2277402; doi:10.1186/1471-2105-9-90)
Supplement: Additional File 4 — T-invariants of the Petri net model of DMD. Description: In the PDF file, DMDTinvariants.pdf, a table, depicting the composition of the t-invariants of the Petri net model of DMD based on the transitions, is provided. [file 1471-2105-9-90-S4.pdf]

Gene regulation of the Duchenne muscular dystrophy

Clustering results: UPGMA

Table 1 - The composition of the t-cluster specific t-invariants based on transitions.

	transition	0	1	6	7	8	10	30	2	5	3	4	9	11	12	13	14	56	57	58	15	17	16	20	21	22	84	18	24	40	42	85	19	25	41	43	86	23	26	28	27	29	31	33	34	32	35	37	36	38	39	59	44	45	47	46	48	49	87	50	51	52	55	53	54	60	61	62	63	67	64	66	65	68	71	75	69	70	76	72	73	74	77	78	79	80	81	82	83
t-cluster 1	t-inv.36																																																								#			#	#																												
t-cluster 2	t-inv.5																																																								#	#	#																													
t-cluster 3	t-inv.34																																									#	#	#	#																																														
t-cluster 4	t-inv.37																																																													#	#	#	#																										
t-cluster 5	t-inv.41																																															#	#																			#														#	#	#	#				
	t-inv.40																																				#											#	#																																			#	#	#				
t-cluster 6	t-inv.39																																													#	#																					#														#	#	#	#				
	t-inv.38																																				#									#	#																																					#	#	#				
t-cluster 7	t-inv.107	#	#	#	#	#	#	#	#	#													#	#	#	#	#												#	#							#	#						#	#	#											#																		#		#
	t-inv.106	#	#	#	#	#	#	#	#	#													#	#	#	#	#												#	#									#	#				#	#	#											#																		#		#
	t-inv.105	#	#	#	#	#	#	#	#	#													#	#	#	#	#														#	#					#	#						#	#	#											#																		#		#
	t-inv.104	#	#	#	#	#	#	#	#	#													#	#	#	#	#														#	#							#	#				#	#	#											#																		#		#
	t-inv.103	#	#	#	#	#	#	#			#	#											#	#	#	#	#												#	#							#	#						#	#	#											#																		#		#
	t-inv.102	#	#	#	#	#	#	#			#	#											#	#	#	#	#												#	#									#	#				#	#	#											#																		#		#
	t-inv.101	#	#	#	#	#	#	#			#	#											#	#	#	#	#														#	#					#	#						#	#	#											#																		#		#
	t-inv.100	#	#	#	#	#	#	#			#	#											#	#	#	#	#														#	#							#	#				#	#	#											#																		#		#
t-cluster 8	t-inv.58	#	#	#	#	#	#	#	#	#			#																									#	#									#	#				#	#	#																																		
	t-inv.57	#	#	#	#	#	#	#	#	#			#																									#	#							#	#						#	#	#																																		
	t-inv.55	#	#	#	#	#	#	#	#	#			#																											#	#							#	#				#	#	#																																		
	t-inv.54	#	#	#	#	#	#	#	#	#			#																											#	#					#	#						#	#	#																																		
	t-inv.52	#	#	#	#	#	#	#			#	#	#																									#	#									#	#				#	#	#																																		
	t-inv.51	#	#	#	#	#	#	#			#	#	#																									#	#							#	#						#	#	#																																		
	t-inv.49	#	#	#	#	#	#	#			#	#	#																											#	#							#	#				#	#	#																																		
	t-inv.48	#	#	#	#	#	#	#			#	#	#																											#	#					#	#						#	#	#																																		
t-cluster 9	t-inv.31	#	#	#	#	#	#	#	#	#																		#	#	#	#	#							#	#							#	#																				#																		#		#
	t-inv.29	#	#	#	#	#	#	#	#	#																		#	#	#	#	#							#	#									#	#																		#																		#		#
	t-inv.27	#	#	#	#	#	#	#	#	#																		#	#	#	#	#									#	#					#	#																				#																		#		#
	t-inv.25	#	#	#	#	#	#	#	#	#																		#	#	#	#	#									#	#							#	#																		#																		#		#
	t-inv.23	#	#	#	#	#	#	#			#	#																#	#	#	#	#							#	#							#	#																				#																		#		#
	t-inv.21	#	#	#	#	#	#	#			#	#																#	#	#	#	#							#	#									#	#																		#																		#		#
	t-inv.19	#	#	#	#	#	#	#			#	#																#	#	#	#	#									#	#					#	#																				#																		#		#
	t-inv.17	#	#	#	#	#	#	#			#	#																#	#	#	#	#									#	#							#	#																		#																		#		#
t-cluster 10	t-inv.95	#	#	#	#	#	#	#	#	#											#	#																	#	#							#	#																				#	#														#						#		#
	t-inv.94	#	#	#	#	#	#	#	#	#											#	#																#	#	#							#	#																				#																		#		#
	t-inv.93	#	#	#	#	#	#	#	#	#											#	#																	#	#									#	#																		#	#														#						#		#
	t-inv.92	#	#	#	#	#	#	#	#	#											#	#																#	#	#									#	#																		#																		#		#
	t-inv.89	#	#	#	#	#	#	#	#	#											#	#																		#	#					#	#																				#	#														#						#		#
	t-inv.88	#	#	#	#	#	#	#	#	#											#	#																#			#	#					#	#																				#																		#		#
	t-inv.87	#	#	#	#	#	#	#	#	#											#	#																		#	#							#	#																		#	#														#						#		#
	t-inv.86	#	#	#	#	#	#	#	#	#											#	#																#			#	#							#	#																		#																		#		#
	t-inv.83	#	#	#	#	#	#	#			#	#									#	#																	#	#							#	#																				#	#														#						#		#
	t-inv.82	#	#	#	#	#	#	#			#	#									#	#																#	#	#							#	#																				#																		#		#
	t-inv.81	#	#	#	#	#	#	#			#	#									#	#																	#	#									#	#																		#	#														#						#		#
	t-inv.80	#	#	#	#	#	#	#			#	#									#	#																#	#	#									#	#																		#																		#		#
	t-inv.77	#	#	#	#	#	#	#			#	#									#	#																		#	#					#	#																				#	#														#						#		#
	t-inv.76	#	#	#	#	#	#	#			#	#									#	#																#			#	#					#	#																				#																		#		#
	t-inv.75	#	#	#	#	#	#	#			#	#									#	#																		#	#							#	#																		#	#														#						#		#
	t-inv.74	#	#	#	#	#	#	#			#	#									#	#																#			#	#							#	#																		#																		#		#
t-cluster 11	t-inv.30	#	#	#	#	#	#	#	#	#																							#	#	#	#	#		#	#							#	#																				#																		#		#
	t-inv.28	#	#	#	#	#	#	#	#	#																							#	#	#	#	#		#	#									#	#																		#																		#		#
	t-inv.26	#	#	#	#	#	#	#	#	#																							#	#	#	#	#				#	#					#	#																				#																		#		#
	t-inv.24	#	#	#	#	#	#	#	#	#																							#	#	#	#	#				#	#							#	#																		#																		#		#
	t-inv.22	#	#	#	#	#	#	#			#	#																					#	#	#	#	#		#	#							#	#																				#																		#		#
	t-inv.20	#	#	#	#	#	#	#			#	#																					#	#	#	#	#		#	#									#	#																		#																		#		#
	t-inv.18	#	#	#	#	#	#	#			#	#																					#	#	#	#	#				#	#					#	#																				#																		#		#
	t-inv.16	#	#	#	#	#	#	#			#	#																					#	#	#	#	#				#	#							#	#																		#																		#		#
t-cluster 12	t-inv.42													#	#	#	#	#	#	#	#	#																								#					#	#		#	#	#						#	#				#																			#			
t-cluster 13	t-inv.33													#	#	#	#	#	#	#																										#			#	#	#	#										#	#																						#	#	#				
	t-inv.32													#	#	#	#	#	#	#																										#	#	#			#	#										#	#																						#	#	#				
t-cluster 14	t-inv.99	#	#	#	#	#	#	#	#	#				#	#	#	#	#	#	#			#	#	#	#	#												#	#						#							#	#	#						#	#				#																				#	#
	t-inv.98	#	#	#	#	#	#	#	#	#				#	#	#	#	#	#	#			#	#	#	#	#														#	#				#							#	#	#						#	#				#																				#	#
	t-inv.97	#	#	#	#	#	#	#			#	#		#	#	#	#	#	#	#			#	#	#	#	#												#	#						#							#	#	#						#	#				#																				#	#
	t-inv.96	#	#	#	#	#	#	#			#	#		#	#	#	#	#	#	#			#	#	#	#	#														#	#				#							#	#	#						#	#				#																				#	#
	t-inv.59	#	#	#	#	#	#	#	#	#				#	#	#	#	#	#	#																			#	#						#							#	#	#						#	#			#																							
	t-inv.56	#	#	#	#	#	#	#	#	#				#	#	#	#	#	#	#																					#	#				#							#	#	#						#	#			#																							
	t-inv.53	#	#	#	#	#	#	#			#	#		#	#	#	#	#	#	#																			#	#						#							#	#	#						#	#			#																							
	t-inv.50	#	#	#	#	#	#	#			#	#		#	#	#	#	#	#	#																					#	#				#							#	#	#						#	#			#																							
	t-inv.91	#	#	#	#	#	#	#	#	#				#	#	#	#	#	#	#	#	#																	#	#						#	#	#			#	#										#	#				#																		#		#
	t-inv.90	#	#	#	#	#	#	#	#	#				#	#	#	#	#	#	#	#	#																	#	#						#			#	#	#	#										#	#				#																		#		#
	t-inv.85	#	#	#	#	#	#	#	#	#				#	#	#	#	#	#	#	#	#																			#	#				#	#	#			#	#										#	#				#																		#		#
	t-inv.84	#	#	#	#	#	#	#	#	#				#	#	#	#	#	#	#	#	#																			#	#				#			#	#	#	#										#	#				#																		#		#
	t-inv.79	#	#	#	#	#	#	#			#	#		#	#	#	#	#	#	#	#	#																	#	#						#	#	#			#	#										#	#				#																		#		#
	t-inv.78	#	#	#	#	#	#	#			#	#		#	#	#	#	#	#	#	#	#																	#	#						#			#	#	#	#										#	#				#																		#		#
	t-inv.73	#	#	#	#	#	#	#			#	#		#	#	#	#	#	#	#	#	#																			#	#				#	#	#			#	#										#	#				#																		#		#
	t-inv.72	#	#	#	#	#	#	#			#	#		#	#	#	#	#	#	#	#	#																			#	#				#			#	#	#	#										#	#				#																		#		#
	t-inv.71	#	#	#	#	#	#	#	#	#				#	#	#	#	#	#	#	#	#																	#	#						#																#	#				#	#														#							#	#
	t-inv.70	#	#	#	#	#	#	#	#	#				#	#	#	#	#	#	#	#	#																#	#	#						#																#	#				#																				#	#
	t-inv.69	#	#	#	#	#	#	#	#	#				#	#	#	#	#	#	#	#	#																	#	#						#					#	#										#	#				#																				#	#
	t-inv.68	#	#	#	#	#	#	#	#	#				#	#	#	#	#	#	#	#	#																			#	#				#																#	#				#	#														#							#	#
	t-inv.67	#	#	#	#	#	#	#	#	#				#	#	#	#	#	#	#	#	#																#			#	#				#																#	#				#																				#	#
	t-inv.66	#	#	#	#	#	#	#	#	#				#	#	#	#	#	#	#	#	#																			#	#				#					#	#										#	#				#																				#	#
	t-inv.65	#	#	#	#	#	#	#			#	#		#	#	#	#	#	#	#	#	#																	#	#						#																#	#				#	#														#							#	#
	t-inv.64	#	#	#	#	#	#	#			#	#		#	#	#	#	#	#	#	#	#																#	#	#						#																#	#				#																				#	#
	t-inv.63	#	#	#	#	#	#	#			#	#		#	#	#	#	#	#	#	#	#																	#	#						#					#	#										#	#				#																				#	#
	t-inv.62	#	#	#	#	#	#	#			#	#		#	#	#	#	#	#	#	#	#																			#	#				#																#	#				#	#														#							#	#
	t-inv.61	#	#	#	#	#	#	#			#	#		#	#	#	#	#	#	#	#	#																#			#	#				#																#	#				#																				#	#
	t-inv.60	#	#	#	#	#	#	#			#	#		#	#	#	#	#	#	#	#	#																			#	#				#					#	#										#	#				#																				#	#
	t-inv.15	#	#	#	#	#	#	#	#	#				#	#	#	#	#	#	#								#	#	#	#	#							#	#						#																#	#				#																				#	#
	t-inv.13	#	#	#	#	#	#	#	#	#				#	#	#	#	#	#	#								#	#	#	#	#									#	#				#																#	#				#																				#	#
	t-inv.11	#	#	#	#	#	#	#			#	#		#	#	#	#	#	#	#								#	#	#	#	#							#	#						#																#	#				#																				#	#
	t-inv.9	#	#	#	#	#	#	#			#	#		#	#	#	#	#	#	#								#	#	#	#	#									#	#				#																#	#				#																				#	#
	t-inv.14	#	#	#	#	#	#	#	#	#				#	#	#	#	#	#	#													#	#	#	#	#		#	#						#																#	#				#																				#	#
	t-inv.12	#	#	#	#	#	#	#	#	#				#	#	#	#	#	#	#													#	#	#	#	#				#	#				#																#	#				#																				#	#
	t-inv.10	#	#	#	#	#	#	#			#	#		#	#	#	#	#	#	#													#	#	#	#	#		#	#						#																#	#				#																				#	#
	t-inv.8	#	#	#	#	#	#	#			#	#		#	#	#	#	#	#	#													#	#	#	#	#				#	#				#																#	#				#																				#	#
t-cluster 15	t-inv.35																																																		|#|#|#|																																				
t-cluster 16	t-inv.47																						#	#	#	#	#																											#	#	#												#																			#			
t-cluster 17	t-inv.7																															#	#	#	#	#																	#	#	#												#																			#			
t-cluster 18	t-inv.44																			#	#																																#	#	#												#	#															#				#			
	t-inv.43																			#	#																#																#	#	#												#																			#			
t-cluster 19	t-inv.6																										#	#	#	#	#																						#	#	#												#																			#			
t-cluster 20	t-inv.46																																																																					#	#					#	#	#	#	#								
	t-inv.45																																																																			#	#							#	#	#	#	#								
	t-inv.4																																																																		#					#				#	#	#	#	#								
t-cluster 21	t-inv.3																																																																		#			#	#		#	#	#	#	#	#										
	t-inv.2																																																																		#	#	#				#	#	#	#	#	#										
	t-inv.1																																																																		#					#	#	#	#	#	#	#										
