# Supplementary material for: Modularization of biochemical networks based on classification of Petri net t-invariants
Source: BMC Bioinformatics. 2008 Feb 8;9:90. doi: 10.1186/1471-2105-9-90 (PMC2277402; doi:10.1186/1471-2105-9-90)
Supplement: Additional File 5 — Clustering results of the Petri net model of DMD. In the ZIP file, DMDClusteringResults.zip, the clustering results of the Petri net model of DMD, using Single Linkage, Complete Linkage, and Neighbor Joining, are provided. For each method, the constructed dendrogram as well as a detailed description of the clustering result is given. [file 1471-2105-9-90-S5.zip › DMDClusteringResults/Neighbor Joining/Clustering results NJ.pdf]

Gene regulation of the Duchenne muscular dystrophy

Clustering results: Neighbor Joining

Using Neighbor Joining the 107 nontrivial t-invariants are split into 21 t-clusters. The biological meaning of the t-invariants belonging to one t-cluster is given below.

t-cluster 1: t-invariants 8 - 15, 60 - 73, 78, 79, 84, 85, 90, 91, 50, 53, 56, 59, 96, 97, 98, 99
DGC downstream pathway, which activates regulated *JNK1*; regulated RAP2B downstream pathway, which activates regulated *NFATc*, followed by transcription and no transcriptional activity, respectively, and deactivation of *NFATc* in nucleus by *JNK1*

t-cluster 2: t-invariants 100 - 107
regulated RAP2B downstream pathway, including Ca release, which activates regulated *NFATc*, followed by a transcription of *MLC2*, *aActin*, *ANF*, and deactivation of *NFATc* in nucleus by regulated CSNK1A1

t-cluster 3: t-invariants 48, 49, 51, 52, 54, 55, 57, 58
regulated RAP2B downstream pathway, including Ca release, which activates regulated *NFATc*; no transcriptional activity due to deactivation of *NFATc* in cytosol by regulated *CSNK1A1*

t-cluster 4: t-invariants 74 - 77, 80 - 83, 86 - 89, 92 - 95
regulated RAP2B downstream pathway, including Ca release, which activates regulated *NFATc*, followed by p21 transcription, and deactivation of *NFATc* in nucleus by regulated *CSNK1A1*

t-cluster 5: t-invariants 17, 19, 21, 23, 25, 27, 29, 31
regulated RAP2B downstream pathway, including Ca release, which activates regulated *NFATc*, followed by transcription of *UTRNA*, and deactivation of *NFATc* in nucleus by regulated *CSNK1A1*

t-cluster 6: t-invariants 16, 18, 20, 22, 24, 26, 28, 30
regulated RAP2B downstream pathway, including Ca release, which activates *NFATc*, followed by transcription of *MYF5*, and deactivation of *NFATc* in nucleus by regulated *CSNK1A1*

t-cluster 7: t-invariant 42
DGC downstream pathway, which activates up-regulated *JNK1*, followed by a *c-JUN* phosphorylation dependent *p21* inhibition; regulated *NFATc* mediates *p21* transcription, followed by a degradation of *NFATc* in nucleus

t-cluster 8: t-invariants 32, 33
DGC downstream pathway, which activates regulated *JNK1*, followed by a *c-JUN* phosphorylation dependent *p21* inhibition; regulation of *CSNK1A1*, which activates *p53*, followed by *p21* transcription

t-cluster 9: t-invariant 37
initiation, up/down-regulation of *JNK1*

t-cluster 10: t-invariant 35
initiation and down-regulation of *NFATc*

t-cluster 11: t-invariant 47
regulated *NFATc* mediates transcription of *MLC2*, *aActin*, and *AFN*, followed by degradation of *NFATc* in nucleus

t-cluster 12: t-invariant 7
regulated *NFATc* mediates transcription of *MYF5*, followed by degradation of *NFATc* in nucleus

t-cluster 13: t-invariants 43, 44
regulated *NFATc* mediates *p21* transcription inhibiting *CDK2*, followed by degradation of *NFATc* in nucleus

t-cluster 14: t-invariant 6
regulated *NFATc* mediates transcription of *UTRNA*, followed by degradation of *NFATc* in nucleus

t-cluster 15: t-invariant 34
initiation of dystrophin followed by generation of *DGC* and simulation of DMD by *DGC* loss

t-cluster 16: t-invariant 36
initiation, down-regulation and removal of calcineurin

t-cluster 17: t-invariant 5
initiation, up-regulation and degradation of calcineurin

t-cluster 18: t-invariants 40, 41
initiation and up-regulation of the gene *CSNK1A1*, the protein *CSNK1A1* activates *p53*, followed by *p21* transcription, which inhibits *CDK2*

t-cluster 19: t-invariants 38, 39
initiation and down-regulation of the gene *CSNK1A1*, the protein *CSNK1A1* activates in a decreased manner *p53* followed by *p21* transcription, which inhibits *CDK2*

t-cluster 20: t-invariants 4, 45, 46
CDK-dependent RB-E2F cell cycle pathway, resulting in transcription of S-phase genes

t-cluster 21: t-invariants 1, 2, 3
RB-E2F cell cycle pathway, inhibited by CDK2-phosphorylated *E2F*
