# Supplementary material for: Modularization of biochemical networks based on classification of Petri net t-invariants
Source: BMC Bioinformatics. 2008 Feb 8;9:90. doi: 10.1186/1471-2105-9-90 (PMC2277402; doi:10.1186/1471-2105-9-90)
Supplement: Additional File 5 — Clustering results of the Petri net model of DMD. In the ZIP file, DMDClusteringResults.zip, the clustering results of the Petri net model of DMD, using Single Linkage, Complete Linkage, and Neighbor Joining, are provided. For each method, the constructed dendrogram as well as a detailed description of the clustering result is given. [file 1471-2105-9-90-S5.zip › DMDClusteringResults/Single Linkage/Clustering results SL.pdf]

Gene regulation of the Duchenne muscular dystrophy

Clustering results: Single Linkage

Using Single Linkage the 107 nontrivial t-invariants are split into 22 t-clusters. The biological meaning of the t-invariants belonging to one t-cluster is given below.

t-cluster 1: t-invariant 36
initiation, down-regulation and removal of calcineurin

t-cluster 2: t-invariant 5
initiation, up-regulation and degradation of calcineurin

t-cluster 3: t-invariant 34
initiation of dystrophin followed by generation of *DGC* and simulation of DMD by *DGC* loss

t-cluster 4: t-invariant 37
initiation, up/down-regulation of *JNK1*

t-cluster 5: t-invariant 35
initiation and down-regulation of *NFATc*

t-cluster 6: t-invariant 41
initiation and up-regulation of the gene *CSNK1A1*, the protein *CSNK1A1* activates *p53*, followed by *p21* transcription, which inhibits *CDK2*

t-cluster 7: t-invariant 40
initiation and up-regulation of the gene *CSNK1A1*, the protein *CSNK1A1* activates *p53*, followed by *p21* transcription, and p21 removal

t-cluster 8: t-invariant 39
initiation and down-regulation of the gene *CSNK1A1*, the protein *CSNK1A1* activates in a decreased manner *p53* followed by *p21* transcription, which inhibits *CDK2*

t-cluster 9: t-invariant 38
initiation and down-regulation of the gene *CSNK1A1*, the protein *CSNK1A1* activates in a decreased manner *p53* followed by *p21* transcription, which inhibits *CDK2*

t-cluster 10: t-invariant 42
DGC downstream pathway, which activates up-regulated *JNK1*, followed by a *c-JUN* phosphorylation dependent *p21* inhibition; regulated *NFATc* mediates *p21* transcription, followed by a degradation of *NFATc* in nucleus

t-cluster 11: t-invariants 32, 33
DGC downstream pathway, which activates regulated *JNK1*, followed by a *c-JUN* phosphorylation dependent *p21* inhibition; regulation of *CSNK1A1*, which activates *p53*, followed by *p21* transcription

t-cluster 12: t-invariants 16 - 31, 74 - 77, 80 - 83, 86 - 89, 92 - 95
regulated RAP2B downstream pathway, including Ca release, which activates regulated *NFATc*, followed by transcription of *UTRNA*, *MYF5* or *p21*, respectively, and deactivation of *NFATc* in nucleus by regulated *CSNK1A1*

t-cluster 13: t-invariants 48, 49, 51, 52, 54, 55, 57, 58, 100 - 107
regulated RAP2B downstream pathway, including Ca release, which activates regulated *NFATc*, deactivation of *NFATc* in nucleus by regulated CSNK1A1

t-cluster 14: t-invariants 8 - 15, 60 - 73, 78, 79, 84, 85, 90, 91, 50, 53, 56, 59, 96, 97, 98, 99
DGC downstream pathway, which activates regulated *JNK1*; regulated RAP2B downstream pathway, which activates regulated *NFATc*, followed by transcription and no transcriptional activity, respectively, and deactivation of *NFATc* in nucleus by *JNK1*

t-cluster 15: t-invariant 47
regulated *NFATc* mediates transcription of *MLC2*, *aActin*, and *AFN*, followed by degradation of *NFATc* in nucleus

t-cluster 16: t-invariant 7
regulated *NFATc* mediates transcription of *MYF5*, followed by degradation of *NFATc* in nucleus

t-cluster 17: t-invariants 43, 44
regulated *NFATc* mediates *p21* transcription inhibiting *CDK2*, followed by degradation of *NFATc* in nucleus

t-cluster 18: t-invariant 6
regulated *NFATc* mediates transcription of *UTRNA*, followed by degradation of *NFATc* in nucleus

t-cluster 19: t-invariant 46
CDK4-dependent RB-E2F cell cycle pathway, resulting in transcription of S-phase genes

t-cluster 20: t-invariant 45
CDK2-dependent RB-E2F cell cycle pathway, resulting in transcription of S-phase genes

t-cluster 21: t-invariant 4
CDK2-dependent RB-E2F cell cycle pathway, phosphorylation of E2F by CDK2, which inhibits RB phosphorylation

t-cluster 22: t-invariants 1, 2, 3
RB-E2F cell cycle pathway, inhibited by CDK2-phosphorylated *E2F*
